# National Summit on Neglected Infections of Poverty in the United States

**DOI:** 10.3201/eid1605.091863

**Published:** 2010-05

**Authors:** Peter Hotez, Eileen Stillwaggon, Marian McDonald, Lynn Todman, Lydia DiGrazia

**Affiliations:** George Washington University, Washington, DC, USA (P. Hotez, L. DiGrazia); Sabin Vaccine Institute, Washington (P. Hotez, L. DiGrazia); Gettysburg College and Eisenhower Institute, Gettysburg, Pennsylvania, USA, and Washington (E. Stillwaggon); Centers for Disease Control and Prevention, Atlanta, Georgia, USA (M. McDonald); Adler School of Professional Psychology, Chicago, Illinois, USA (L. Todman)

**Keywords:** Poverty, cysticercosis, congenital cytomegalovirus, Chagas disease, toxocariasis, toxoplasmosis, trichomoniasis, parasites, United States, conference summary

The first-ever National Summit on Neglected Infections of Poverty in the United States was held October 27, 2009, in Washington, DC, bringing together key leaders in public health, public policy, and government. Organized by the Eisenhower Institute, the George Washington University, the Adler School, the American Public Health Association, and the Centers for Disease Control and Prevention, the goal of the summit was to raise awareness about neglected infections of poverty in the United States and identify resources and actions to address them.

Neglected infections of poverty are a group of chronic and debilitating parasitic and other infections (including congenital infections) that disproportionately affect people living in poverty. Major neglected infections of poverty in the United States include toxocariasis, trichomoniasis, toxoplasmosis, cysticercosis, Chagas disease, and congenital cytomegalovirus infection (*1*,*2*). Neglected infections of poverty tend to be concentrated in areas of extreme poverty, including post-Katrina Louisiana and the Mississippi Delta, the border with Mexico, Appalachia, tribal lands, and disadvantaged urban areas, where these diseases perpetuate poverty because of their adverse health impact on child development, pregnancy, and worker productivity (*1*).

Specific objectives of the summit were to highlight knowledge about the impact of neglected infections of poverty and their modes of transmission. The summit participants also aimed to identify gaps in surveillance, diagnostics, prevention and management strategies, as well as to build collaboration with diverse stakeholders to prioritize national efforts to address these infections.

The summit’s program included leading experts on the epidemiology and clinical aspects of neglected infections of poverty, as well as on the economic and social determinants of health disparities in the United States. These diseases of poverty are of strategic importance for the populations affected, as are neglected tropical diseases that are endemic to developing countries. Both kinds of infections result in considerable illness and adverse birth outcomes, impair child development and cognition, adversely affect worker productivity, and are important causes of preventable disability and death. The disease burden of neglected tropical diseases measured in disability adjusted life-years is roughly equivalent to that of HIV/AIDS, malaria, or tuberculosis in low- and middle-income countries. Data on the disease impact of neglected infections of poverty in the United States are currently limited, suggesting an urgent need for enhanced surveillance and disease impact assessments for affected populations.

An important focus of the summit and a key aspect of the epidemiology of infections of poverty in the United States is their geographic concentration in impoverished areas, as well as their high prevalence among people who are poor and among racial and ethnic minorities, women, and children. For example, cysticercosis is a leading cause of epilepsy and emergency room visits among poor children, especially among Hispanic Americans living in the West and Southwest. Among young adults living in these same areas, Chagas disease can result in severe cardiomyopathy and early death. Toxocariasis, a larval helminthiasis believed to be affecting millions of African Americans, has been linked to asthma and developmental delays, while trichomoniasis is a one of the most common sexually transmitted infections among African-American women. Because healthcare providers receive little or no training on these conditions, the neglected infections of poverty often go undiagnosed, and there is an urgent need for better disease prevalence estimates in the major affected areas. Moreover, the economic toll from these infections may also be substantial because they cause poor school performance, young adult disability, premature death, and hospitalization; in some cases, the costs of therapy are also high because correct diagnosis is delayed.

Summit participants discussed the following Action Steps: 1) improve understanding of the impact and geographic distribution of neglected infections of poverty; 2) enhance understanding of modes of transmission and human ecology of infections of poverty; 3) enhance prevention and promote research and development; and 4) disseminate information to multiple audiences. Through a wide-ranging and rich dialogue, summit participants developed 4 priority areas for follow-up. These areas will be pursued by working groups convening in early 2010.

- Outreach and mobilization
- Data and surveillance needs
- Economic impact of neglected infections of poverty
- Research and development needs

For further information see http://inside.gwumc.edu/niops/ or contact neglectedinfectionsofpoverty@cdc.gov
